# Supplementary material for: Identification of splice regulators of fibronectin-EIIIA and EIIIB by direct measurement of exon usage in a flow-cytometry based CRISPR screen
Source: Sci Rep. 2021 Oct 6;11:19835. doi: 10.1038/s41598-021-99079-1 (PMC8494765; doi:10.1038/s41598-021-99079-1)
Supplement: Supplementary file 1 — Supplementary Information 1. [file 41598_2021_99079_MOESM1_ESM.pdf]

<u>Prime Flow Staining Protocol, adapted from ThermoFisher RNA Assay</u>

> *Note: This procedure is written based on the use of the 1.5-mL tubes provided in the kit throughout the assay. The use of these tubes is important during the hybridization and signal amplification steps to control residual volumes. However, staining with antibodies and/or fixable viability dyes as well as fixation and permeabilization (Steps 1–16) may be done in bulk and in any tube desired. If these procedures are done in bulk, use volumes such that cells do not exceed 1 x 10^7 cells/mL. To perform the assay in 96-well plates, refer to Appendix A7 of this user manual.*

Day 1: Antibody staining, fixation, and permeabilization

1. Pre-warm PrimeFlow RNA Wash Buffer to room temperature. This buffer will first be used in Step 13.
2. Aliquot **1–5 x 10^6 cells** in Flow Cytometry Staining Buffer into the 1.5-mL tubes provided in the kit.
5. <u>Prepare Fixation Buffer 1</u> by mixing equal parts of PrimeFlow RNA Fixation Buffer 1A and PrimeFlow RNA Fixation Buffer 1B. Mix gently by inverting.

    Note:You will need 1 mL of this buffer per sample. Prepare this buffer in bulk to accommodate all samples. Avoid vortexing or vigorously shaking this buffer. This buffer should be prepared fresh. Dispose of any unused buffer.

6. **Add 1 mL of prepared Fixation Buffer 1** to each sample and invert to mix. **Incubate for 30 minutes at 2–8°C**.
7. **Spin down at 800 x g for 5 minutes**. Discard supernatant and resuspend cells in the residual volume.
8. <u>Prepare 1X PrimeFlow RNA Permeabilization Buffer</u> with RNase Inhibitors by diluting PrimeFlow RNA Permeabilization Buffer (10X) to 1X with RNase-free water. Then add RNase Inhibitor 1 (1,000X) at a 1/1,000 dilution and RNase Inhibitor 2 (100X) at a 1/100 dilution. Mix gently by inverting. Keep at 2–8°C.

    Note:You will need 3 mL of this buffer per sample. Prepare this buffer in bulk to accommodate all samples. Avoid vortexing or vigorously shaking this buffer. This buffer should be prepared fresh. Dispose of any unused buffer.

9. **Add 1 mL of 1X PrimeFlow RNA Permeabilization Buffer with RNase Inhibitors to each sample**, invert to mix, and spin down at 800 x g for 5 minutes, then discard supernatant and resuspend cells in the residual volume.
10. Repeat Step 9.
13. <u>Prepare 1X PrimeFlow RNA Fixation Buffer 2</u> by combining 125 µL of PrimeFlow RNA Fixation Buffer 2 (8X) with 875 µL of PrimeFlow RNA Wash Buffer per sample. Mix gently by inverting.

    Note: You will need 1 mL of this buffer per sample. Prepare this buffer in bulk to accommodate all samples. For example, for 10 samples, combine 1.25 mL of PrimeFlow RNA Fixation Buffer 2 (8X) with 8.75 mL of PrimeFlow Wash Buffer. Avoid vortexing or vigorously shaking this buffer. This buffer should be prepared fresh. Dispose of any unused buffer.

14. **Add 1 mL of 1X PrimeFlow RNA Fixation Buffer 2 to each sample** and invert to mix, and then **incubate for 60 minutes in the dark at room temperature**.

    Note: It is important to fix the samples at room temperature. Do not perform this step on ice.

16. Spin down at 800 x g for 5 minutes, then aspirate all but 100 µL of supernatant and resuspend cells in the residual volume by vortexing gently.

    Note: If staining, fixation, and permeabilization were performed in bulk, the cells should be transferred into the 1.5-mL tubes provided in the kit during the following wash steps and before storage overnight.

17. **Add 1 mL of PrimeFlow RNA Wash Buffer to each sample**, invert to mix, and spin down at 800 x g for 5 minutes, then aspirate all but 100 µL of supernatant and resuspend cells in the residual volume by vortexing gently.
18. Repeat Step 17.

    Note: It is critical that the residual volume is as close to 100 µL as possible. Use the markings on the 1.5-mL tubes provided in the kit to assist.

Day 1: Target probe hybridization

Note:It is critical that the residual volume after all washes be as close to 100 µL as possible. Use the markings on the 1.5-mL tubes provided in the kit to assist.

Note:Diluted Target Probes should be pipetted directly into the 100 µL of residual volume and samples should be mixed well before incubating. Do not pipette solutions onto the walls of the tubes.

20.Thaw Target Probes, including Positive Control Target Probe Sets (20X), at room temperature.

21. Pre-warm PrimeFlow RNA Target Probe Diluent to 40°C.

22. Dilute Positive Control Target Probe Sets (20X) 1/20 in PrimeFlow RNA Target Probe Diluent. Mix thoroughly by pipetting up and down.

Note:You will need 100 µL of diluted Target Probes for each sample. If you are adding more than one Target Probe per sample, adjust the volume of the PrimeFlow RNA Target Probe Diluent accordingly so that the final volume remains 100 µL per sample.

23. **Add 100 µL of diluted Target Probe(s) directly into the cell suspension** for the appropriate samples and briefly vortex to mix, and then incubate for 2 hours at 40°C. Invert samples to mix after 1 hour.

24. **Add 1 mL of PrimeFlow RNA Wash Buffer** to each sample, invert to mix, and spin down at 800 x g for 5 minutes. Aspirate all but 100 µL of supernatant and resuspend cells in the residual volume by vortexing gently.

25. <u>Prepare PrimeFlow RNA Wash Buffer with RNase Inhibitor 1</u> by adding RNase Inhibitor 1 (1,000X) at a 1/1,000 dilution to the PrimeFlow RNA Wash Buffer. Mix gently by inverting.

Note:You will need 1 mL of this buffer per sample. Prepare this buffer in bulk to accommodate all samples. This buffer should be prepared fresh. Dispose of any unused buffer.

26. **Add 1 mL of PrimeFlow RNA Wash Buffer with RNase Inhibitor 1** to each sample, invert to mix, and spin down at 800 x g for 5 minutes. Aspirate all but 100 µL of supernatant and resuspend cells in the residual volume by vortexing gently.

<u>Stopping point</u>

27. Store samples overnight in the dark at 2–8°C.

Note:We recommend this stopping point for ease-of-use and a more manageable workflow. However, if desired, Step 27 may be skipped. If skipping this step, proceed to Step 28 and continue through to the end of the protocol

Day 2: Signal amplification

Note:It is critical that the residual volume after all washes be as close to 100 µL as possible. Use the markings on the 1.5-mL tubes provided in the kit to assist.

Note:PrimeFlow RNA PreAmp Mix, PrimeFlow RNA Amp Mix, and diluted Label Probes should be pipetted directly into the 100 µL of residual volume and samples should be mixed well before incubating. Do not pipette these solutions onto the walls of the tubes.

28. Pre-warm samples and PrimeFlow RNA Wash Buffer to room temperature.

29. Pre-warm PrimeFlow RNA PreAmp Mix, PrimeFlow RNA Amp Mix, and PrimeFlow RNA Label Probe Diluent to 40°C.

30. Thaw PrimeFlow RNA Label Probes (100X) on ice in the dark.

Note:This can be done during the Amp Mix incubation (Step 34).

31. **Add 100 µL of PrimeFlow RNA PreAmp Mix** directly into the cell suspension for each sample and briefly vortex to mix. Incubate for 1.5 hours at 40°C.

32. **Add 1 mL of PrimeFlow RNA Wash Buffer** to each sample, invert to mix, and spin down at 800 x g for 5 minutes. Aspirate all but 100 µL of supernatant and resuspend cells in the residual volume by vortexing gently.

33. Repeat Step 32 two times, for a total of three washes.

34. **Add 100 µL of PrimeFlow RNA Amp Mix** directly into the cell suspension for each sample and briefly vortex to mix. Incubate for 1.5 hours at 40°C.

35. **Add 1 mL of PrimeFlow RNA Wash Buffer** to each sample, invert to mix, and spin down at

800 x g for 5 minutes. Aspirate all but 100 µL of supernatant and resuspend cells in the residual volume by vortexing gently.

36. Repeat Step 35.

37. Dilute PrimeFlow RNA Label Probes (100X) 1/100 in PrimeFlow RNA Label Probe Diluent.

Note:You will need 100 µL of diluted Label Probes for each sample. Prepare diluted Label Probes in bulk to accommodate all samples.

38. **Add 100 µL of diluted Label Probes** directly into the cell suspension for each sample and briefly vortex to mix, and then incubate for 1 hour at 40°C.

39. **Add 1 mL of PrimeFlow RNA Wash Buffer** to each sample, invert to mix, and spin down at 800 x g for 5 minutes. Aspirate all but 100 µL of supernatant and resuspend cells in the residual volume by vortexing gently.

40. Repeat Step 39.

41. **Add 1 mL of PrimeFlow RNA Storage Buffer** or Flow Cytometry Staining Buffer to each sample, invert to mix, and spin down at 800 x g for 5 minutes. Aspirate all but 100 µL of supernatant and resuspend cells in the residual volume by vortexing gently.

42. Transfer samples to 12 x 75 mm polystyrene tubes, resuspend in an appropriate volume of PrimeFlow RNA Storage Buffer or Flow Cytometry Staining Buffer, and analyze samples on a flow cytometer.

Note: Samples may be stored before analysis. If samples have been stained with antibodies conjugated to tandem dyes, we recommend storing the samples in IC Fixation Buffer by mixing 100 µL of cells with 100 µL of IC Fixation Buffer. Store samples in the dark at 2–8°C for up to three days

Prime Flow Target Probe Oligos:

ACCESSION	NAME	Type	Fn1 PROBE REGION	SEQUENCE	TM	LENGTH
Constitutive FN probe set						
NM_010233	Fn11-4R	LE	2655-2679bp	tgctgtagtctgtgaggtagacagg	64.89	25
NM_010233	Fn12-4R	LE	2680-2698bp	ggtctggaggagcgtcagg	63.34	19
NM_010233	Fn13-4R	LE	2699-2719bp	catcaacctggtccacggtag	63.93	21
NM_010233	Fn14-4R	LE	2720-2742bp	ccaccgaacaacaatggaagtat	65.19	23
NM_010233	Fn15-4R	LE	2743-2764bp	tgataggtgcctggggtctact	64.51	22
NM_010233	Fn16-4R	LE	2765-2791bp	aaggtgaatagacaattctataccctg	64.57	27
NM_010233	Fn17-4R	LE	2792-2815bp	gctctgtgctactgccttctactg	65.07	24
NM_010233	Fn18-4R	LE	2816-2834bp	gccgtttcagggaggttga	64.18	19
NM_010233	Fn19-4R	LE	2835-2854bp	cgctgagggtgacggagttg	67.24	20
NM_010233	Fn110-4R	LE	2855-2872bp	gaacaccgggctgcaggt	64.53	18
NM_010233	Fn111-4R	LE	2873-2899bp	ccacagcatagatagtgatgttgtact	64.13	27
NM_010233	Fn112-4R	LE	2900-2921bp	ggtgtgctctcctggttctcct	66.09	22
NM_010233	Fn113-4R	LE	2922-2943bp	ggtctcttgttggatgaaaacg	63.09	22
NM_010233	Fn114-4R	LE	2944-2963bp	tcagatcttggggtgccagt	63.5	20
NM_010233	Fn115-4R	BL	2964-2981bp	gtcggaggggggacgtta	63.66	18
NM_010233	Fn116-4R	LE	2982-3005bp	gtcagttccacaaactgtaggtcc	63.92	24
NM_010233	Fn117-4R	LE	3006-3029bp	cacatgatggtgactttcacatca	64.51	24
NM_010233	Fn118-4R	LE	3030-3051bp	caccacactatcaggaggggtc	64.59	22
NM_010233	Fn119-4R	LE	3052-3072bp	gacctccacacggtatccaga	63.17	21
NM_010233	Fn120-4R	BL	3073-3090bp	gggcaggctgacaggcag	65.16	18
NM_010233	Fn121-4R	BL	3091-3108bp	cctctgcccatgttcccc	63.81	18
NM_010233	Fn122-4R	LE	3109-3132bp	aaaggtatttctgttgacaggcag	64.5	24
NM_010233	Fn123-4R	LE	3133-3153bp	ggacagcccagtgatttcagc	65.72	21
NM_010233	Fn124-4R	LE	3154-3174bp	gaagaggtacgtgaccccagg	64.78	21
NM_010233	Fn125-4R	LE	3175-3196bp	cctggtgcacagcaaagacttt	66.06	22
NM_010233	Fn126-4R	LE	3197-3216bp	cagaggattgctttccctgc	63.6	20
NM_010233	Fn127-4R	LE	3217-3236bp	ttggtggtctgttgtgccgt	65.21	20
NM_010233	Fn128-4R	LE	3237-3257bp	aggttagtgggagcgtcgagt	64.5	21
NM_010233	Fn129-4R	LE	3258-3281bp	ctgtcagtttcattgacaaactgg	63.47	24
NM_010233	Fn130-4R	LE	3282-3305bp	ggagtccacgttaccagaactgtt	65.96	24
NM_010233	Fn131-4R	LE	3306-3325bp	ctgctatacgggctcgaggt	63.58	20
NM_010233	Fn132-4R	LE	3326-3343bp	ccgcggtcagtcggtagc	65.53	18
NM_010233	Fn133-4R	LE	3344-3360bp	gcctcctcgggtcaggc	63.81	17
NM_010233	Fn134-4R	BL	3361-3381bp	cacattgtactgcttgggctg	62.93	21
NM_010233	Fn135-4R	BL	3382-3399bp	cttggaggccaagggtcc	63.31	18
NM_010233	Fn136-4R	LE	3400-3421bp	gctgcagatttctcaggggata	65.28	22
NM_010233	Fn137-4R	LE	3422-3443bp	gtcacggtgtactcagacccag	63.05	22

NM_010233	Fn138-4R	BL	3444-3463bp	tccctttcacagccaccaag	64.4	20
NM_010233	Fn139-4R	BL	3464-3483bp	ggctttgggactctgctggt	65.26	20
NM_010233	Fn140-4R	LE	3484-3505bp	gcagggtagtaaagactccggt	63.89	22
NM_010233	Fn141-4R	LE	3506-3524bp	ggaatggagcgcagaggct	66.17	19
NM_010233	Fn142-4R	LE	3525-3546bp	tgtcacctcggtgttgtaaggt	63.39	22
NM_010233	Fn143-4R	LE	3547-3569bp	caggtgatcacaattgtggtctc	63.17	23
NM_010233	Fn144-4R	BL	3570-3586bp	tccttggagcgggggtc	64.16	17
NM_010233	Fn145-4R	LE	3587-3606bp	tacacccagcttgaagccaa	62.88	20
NM_010233	Fn146-4R	LE	3607-3623bp	cctccctggctcggtcg	64.83	17
NM_010233	Fn147-4R	LE	3624-3642bp	cacttctcggggtgcctca	64.78	19
NM_010233	Fn148-4R	LE	3643-3665bp	acaatgctcccagagtctgaagt	63.82	23
EIIIB probe set						
ACCESSION	NAME	TYPE	PROBE REGION	SEQUENCE	TM	LENGTH
NM_010233	Fn11-1R	LE	4047-4065bp	gtcagtgagctggggcacc	63.97	19
NM_010233	Fn12-1R	LE	4066-4093bp	ttgaatcagttatatcaacaaagcttag	64.19	28
NM_010233	Fn13-1R	LE	4094-4111bp	tccacctcaggccgatgc	65.29	18
NM_010233	Fn14-1R	LE	4112-4131bp	ggtggaagagtttagcgggg	65.27	20
NM_010233	Fn15-1R	LE	4132-4154bp	actgtgattcggtacccgataat	64.16	23
NM_010233	Fn16-1R	LE	4155-4173bp	cccttctcctgccgcaact	66.17	19
NM_010233	Fn17-1R	LE	4174-4198bp	ccacaaaatcttcaaaaatagggat	65.56	25
NM_010233	Fn18-1R	LE	4199-4227bp	tgtaactgtgtagtatcctactgaggagt	64.35	29
NM_010233	Fn19-1R	LE	4228-4243bp	tgccaggctccagccc	63.18	16
NM_010233	Fn110-1R	LE	4244-4268bp	gtgataacgctgatgtcatagtcaa	63.54	25
NM_010233	Fn111-1R	LE	4269-4291bp	cactctctccgccattaatgaga	65.47	23
NM_010233	Fn112-1R	LE	4292-4314bp	ttgctgtgtcagtgtagtagggg	63.7	23
EIIIA probe set						
ACCESSION	NAME	TYPE	PROBE REGION	SEQUENCE	TM	LENGTH
NM_010233	Fn11-1R	LE	5415-5436bp	cagtcctttagggcgatcaatg	65.76	22
NM_010233	Fn12-1R	LE	5437-5458bp	cgacatccacatcagtgaatgc	64.7	22
NM_010233	Fn13-1R	LE	5459-5479bp	cccaagcaattttgatggaat	63.58	21
NM_010233	Fn14-1R	LE	5480-5497bp	cttgcccctgtgggcttt	64.24	18
NM_010233	Fn15-1R	LE	5498-5519bp	taggtcaccctgtacctggaaa	63.01	22
NM_010233	Fn16-1R	LE	5520-5538bp	tccatcctcagggctcgag	63.93	19
NM_010233	Fn17-1R	LE	5539-5557bp	cagggaaaagctcccggat	65.28	19
NM_010233	Fn18-1R	LE	5558-5579bp	gtgtcgtcttcaccatcaggtg	63.66	22
NM_010233	Fn19-1R	LE	5580-5596bp	ggccctgcagctctgca	63.83	17
NM_010233	Fn110-1R	LE	5597-5614bp	actcagaccccggcctga	63.84	18
NM_010233	Fn111-1R	LE	5615-5636bp	aaggcaaccacactgactgtgt	63.17	22
NM_010233	Fn112-1R	LE	5637-5657bp	tggctctccatatcatcgtgc	64.6	21
