# Supplementary material for: Identification of splice regulators of fibronectin-EIIIA and EIIIB by direct measurement of exon usage in a flow-cytometry based CRISPR screen
Source: Sci Rep. 2021 Oct 6;11:19835. doi: 10.1038/s41598-021-99079-1 (PMC8494765; doi:10.1038/s41598-021-99079-1)
Supplement: Supplementary file 2 — Supplementary Information 2. [file 41598_2021_99079_MOESM2_ESM.pdf]

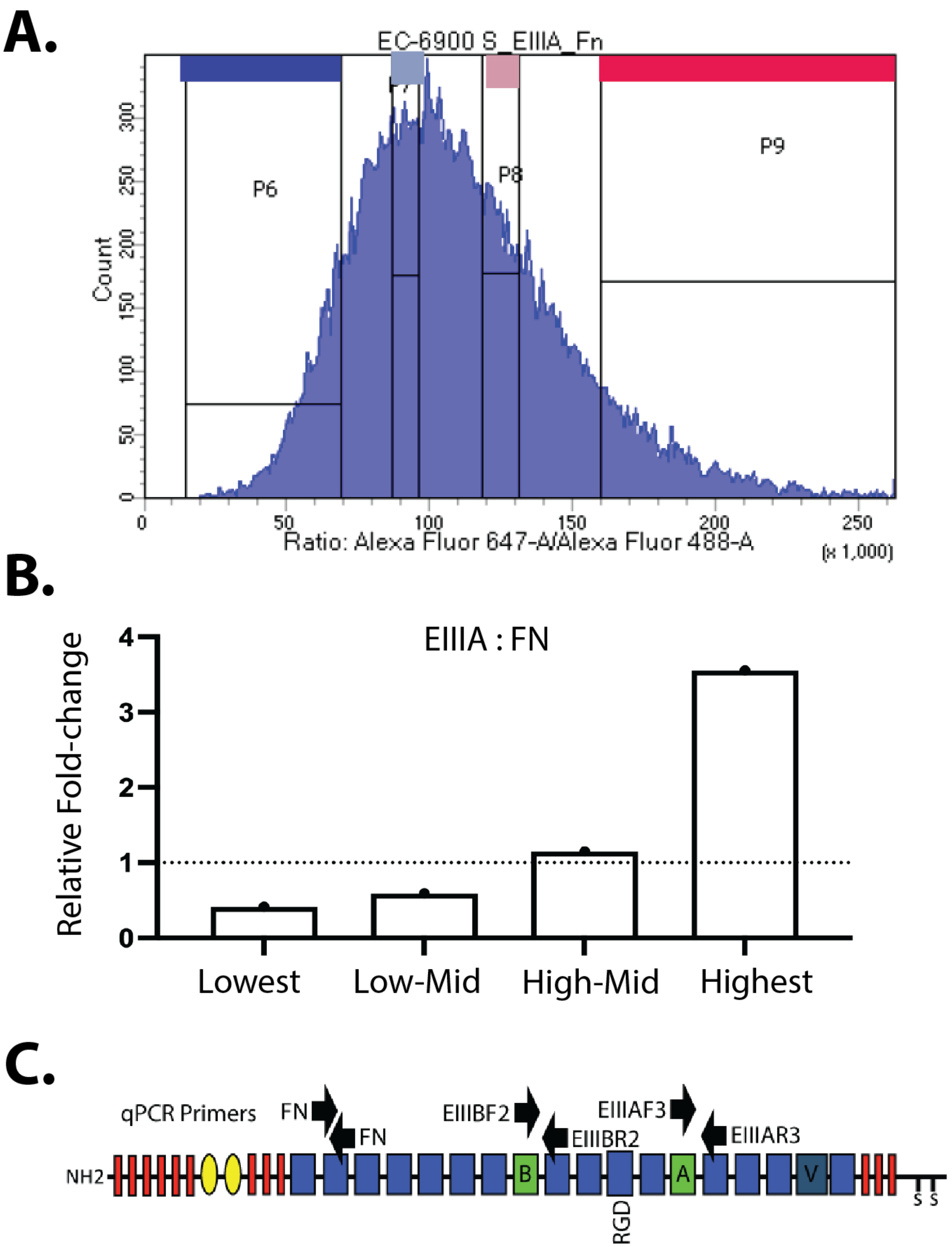

SI Figure 1. Orthoganol assessment of EIIIA inclusion levels in sorted cells by quantitative PCR. (A) Aortic endothelial cells were stained for FN and EIIIA, and sorted into four groups, based on the ratio of EIIIA to total FN (colored bars at top of A). (B) Isolated groups were assessed by quantitative PCR for the ration of EIIIA to total FN. (C) The location of qPCR primers chosen is shown, as previously reported (Murphy et al. 2014 ATVB).

A.

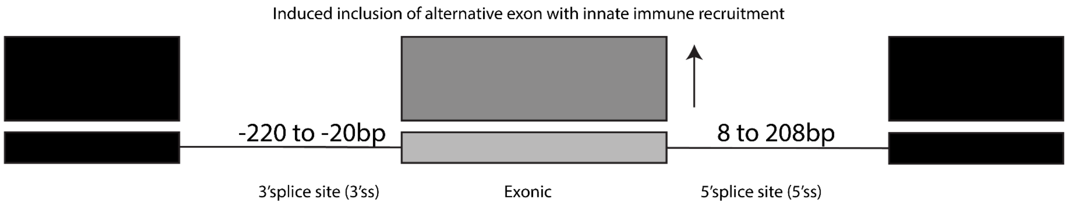

Induced-3'ss	FDR q-val	FWER p-val
RBM4B ENSMUSG00000056951	0.001186047	0.001
CPEB4 CPEB3	5.93E-04	0.001
U2AF2	0.001177889	0.003
SRSF7 (human)	8.83E-04	0.003
CPEB2	7.07E-04	0.003
PPRC1	7.82E-04	0.004
ANKHD1 ANKRD17 ENSMUSG00000089	0.005609915	0.035
CNOT4	0.010228904	0.07
RALY	0.01151817	0.087
ZCRB1	0.010604077	0.089
PTBP1 PTBP2 ROD1	0.009765377	0.09
RBM8A B020018G12RIK	0.008951596	0.09
RALYL HNRNPC	0.00852869	0.093
ZC3H10	0.008156597	0.096
ELAVL1	0.007690097	0.097

Induced-exonic	FDR q-val	FWER p-val
RBM4B ENSMUSG00000056951	8.33E-04	0.001
DAZAP1	4.17E-04	0.001
PPRC1	2.78E-04	0.001
SRSF7 (human)	2.08E-04	0.001
SF3B4	0.001634647	0.01
SRSF2	0.001472269	0.011
RBMS3	0.001261945	0.011
ENSMUSG00000021927 HNRNPA1 HNRNPA3 GM8	0.001600816	0.016
RBM8A B020018G12RIK	0.00192553	0.022
SRSF10 SRSF12	0.004452667	0.055

Induced-5'ss	FDR q-val	FWER p-val
CELF3	0	0
EIF2S1	0	0
CELF4	0	0
RBFOX1 RBFOX3 RBFOX2	0	0
ZCRB1	0	0
CELF6	3.46E-04	0.002
RBM4B ENSMUSG0000005695	2.97E-04	0.002
HNRNPK GM7964	0.001803027	0.014
SRSF7 (human)	0.001836342	0.016
ELAVL1	0.001871341	0.018
PCBP1	0.001980323	0.021
ANKHD1 ANKRD17 ENSMUSG	0.002096575	0.024
U2AF2	0.003849339	0.046
PCBP3 PCBP4	0.005633013	0.071
PPRC1	0.006057801	0.082

B.

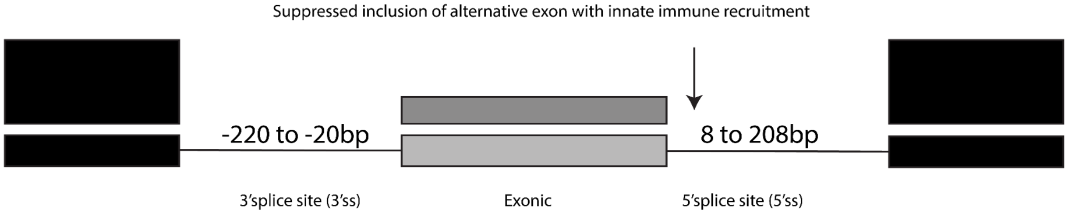

Suppressed-3'ss	FDR q-val	FWER p-val
U2AF2	0	0
RALY	0	0
ELAVL1	0	0
CPEB4 CPEB3	0	0
TIAL1 TIA1	0	0
RALYL HNRNPC	0	0
PTBP1 PTBP2 ROD1	0	0
CELF3	0	0
RBM4B ENSMUSG00000056951	0	0
PCBP3 PCBP4	1.29E-04	0.001
RBM24	2.13E-04	0.002
PPRC1	4.91E-04	0.005
CELF4	4.53E-04	0.005
PCBP1	4.21E-04	0.005
HUMAN-SRSF7	0.002085737	0.027
CPEB2	0.002856554	0.039
RBM38	0.003806701	0.054
ANKHD1 ANKRD17 ENSMUSG00000089	0.004778821	0.072
RBM42	0.006076762	0.095

Suppressed-exonic	FDR q-val	FWER p-val
PABPN1	0	0
CNOT4	0	0
YBX1 CSDA	0.001006892	0.003
SART3	0.001494747	0.006
PABPC1 PABPC6 PABPC2	0.001815933	0.009
HNRPLL	0.005136305	0.031
SRSF10 SRSF12	0.004815856	0.034
HNRNPL	0.008012875	0.062
SRSF1 ENSMUSG00000090049	0.011674899	0.1

Suppressed-5'ss	FDR q-val	FWER p-val
CELF4	0.001309524	0.001
RALY	0.003265298	0.005
TARDBP	0.002176865	0.005
CPEB2	0.001945149	0.006
CELF3	0.001556119	0.006
U2AF2	0.001296766	0.006
RBM24	0.001974098	0.011
HUMAN-SRSF7	0.002744222	0.018
PTBP1 PTBP2 ROD1	0.002841946	0.021
ELAVL1	0.003155093	0.026
TIAL1 TIA1	0.002962607	0.027
SART3	0.002805592	0.028
CELF6	0.002973515	0.032
RBM45	0.003462037	0.04
PABPC1 PABPC6 PABPC2	0.005322549	0.065
CPEB4 CPEB3	0.005838832	0.076
PABPC5	0.006703992	0.092

SI Figure 2. Identification of splice factors active in the response of the endothelium to low and disturbed flow. Motif enrichment analysis for binding sites of splice factors (from CisBP-RNA database) using GSEA, as described in Murphy et al., 2018 eLife. The top 30 6-mer binding sites for each RNAbp were used to determine enrichment of these RNAbp specific motifs among 6-mers enriched in the selected regions in regulated exons versus non-regulated exons. (A) Analysis of regulated skipped exons induced by low flow and macrophage recruitment. (B) Analysis of regulated skipped exons suppressed by low flow and macrophage recruitment. Top ranking RNAbp from this list were combined with differentially expressed RNAbp and additional known regulators of EIIIA and EIIIB (e.g. Srsf5) to generate the list of target RNAbp for the small-scale screen.

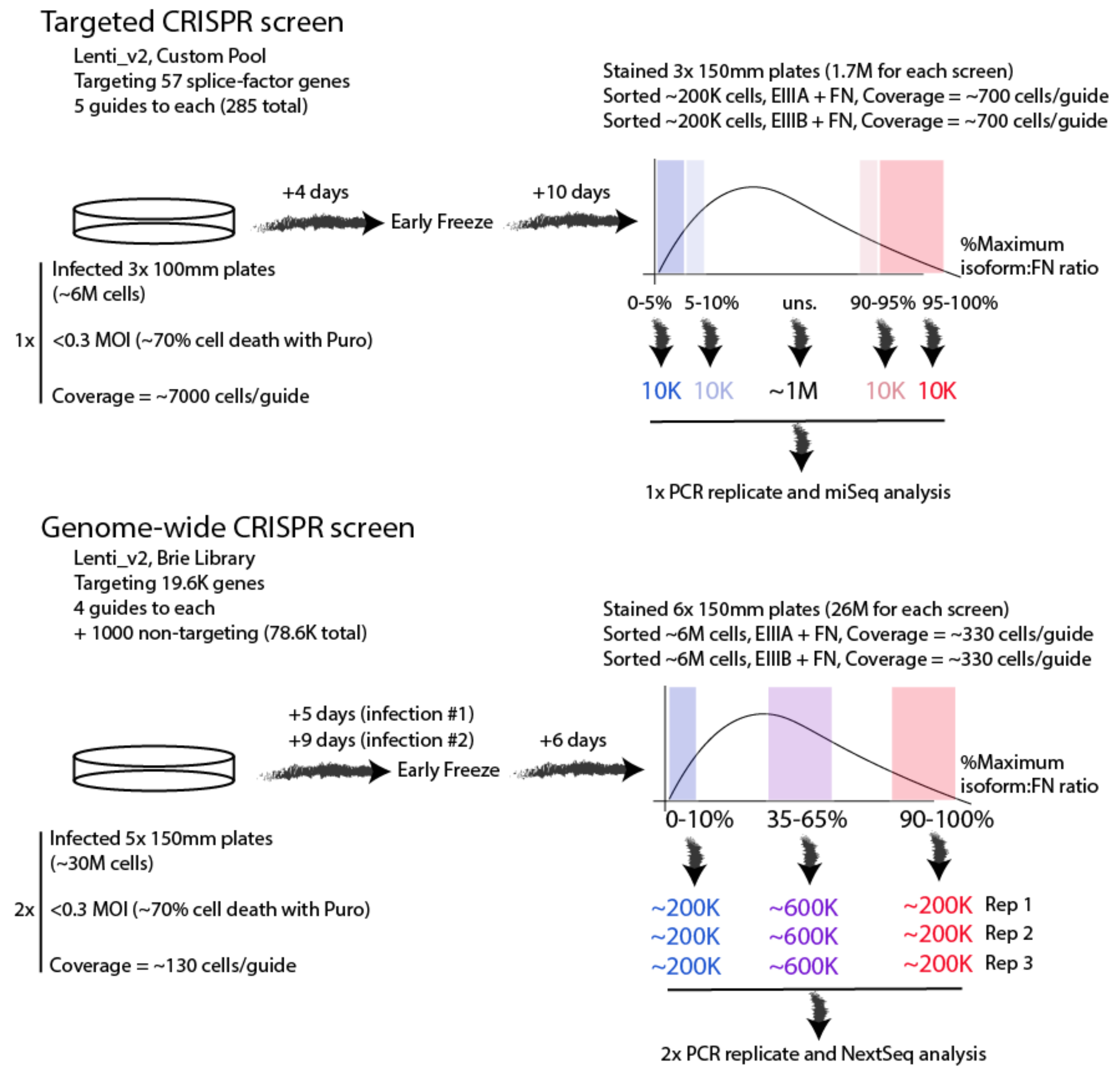

SI Figure 3. Outline of CRISPR screening approaches. For both the targeted screen and the larger scale genome-wide screen, cells were infected with virus derived from lentivirus expressing the indicated libraries at <0.3 MOI and selected under puromycin for stable integration of lentiviral constructs. Cell cultures were kept at sufficient size to maintain a coverage of ~7000 cells per guide for the targeted screen and ~100 cells per guide for the genome-wide screen. For the genome-wide screen, this was done in two separate infections, which were then pooled. Within ~2 weeks of infection, cells were stained for EIIIA and FN, or EIIIB and FN, according to the PrimeFlow protocol. The indicated bins were sorted, based on the ratio of the isoform specific (EIIIA or EIIIB) probes to the total FN probe. For the genome-wide screen, several replicates of each bin were obtained, for biological replicates. For the genome-wide screen, the PCR of insertions was performed in two separate PCR replicates, to control for PCR error.

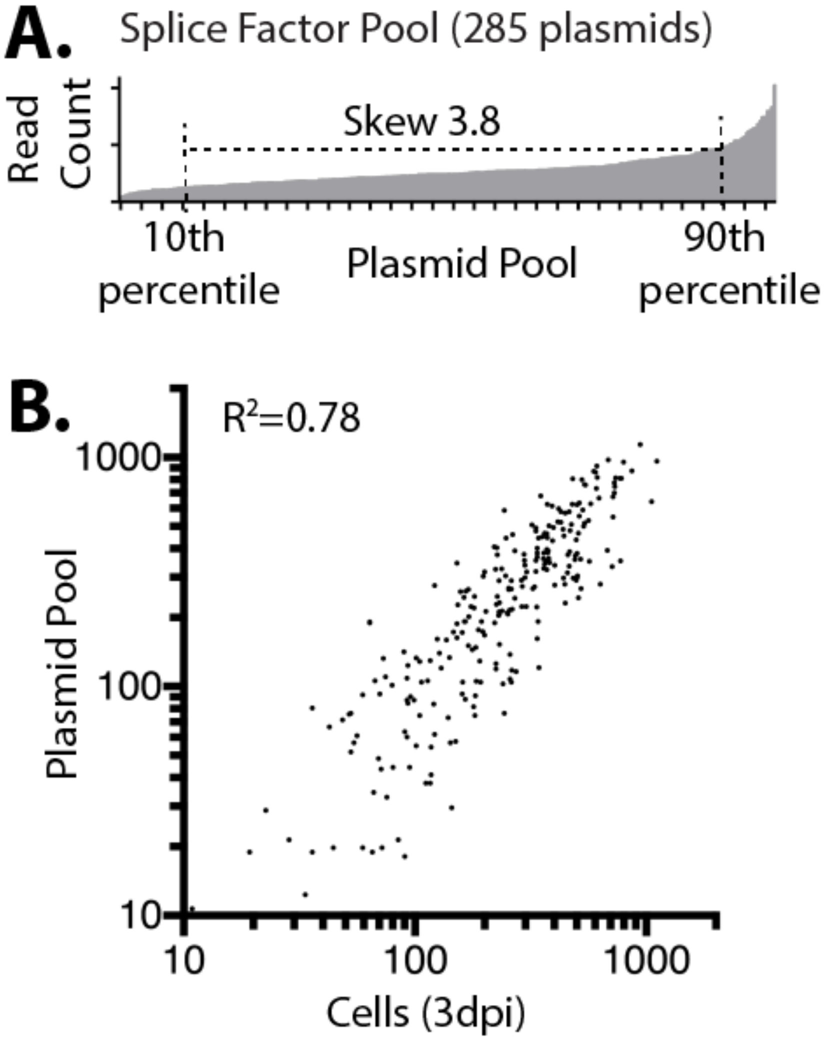

SI Figure 4. Targeted pool representation in cells. (A) Read density (y-axis) for each guide construct in the plasmid library for a list of 57 candidate splice factors (5 guides for each gene, x-axis). (B) Read count for the plasmid pool (A, y axis) and cells after infection with lentivirus derived from the plasmid pool (x axis). Each point represents a single gRNA sequence.

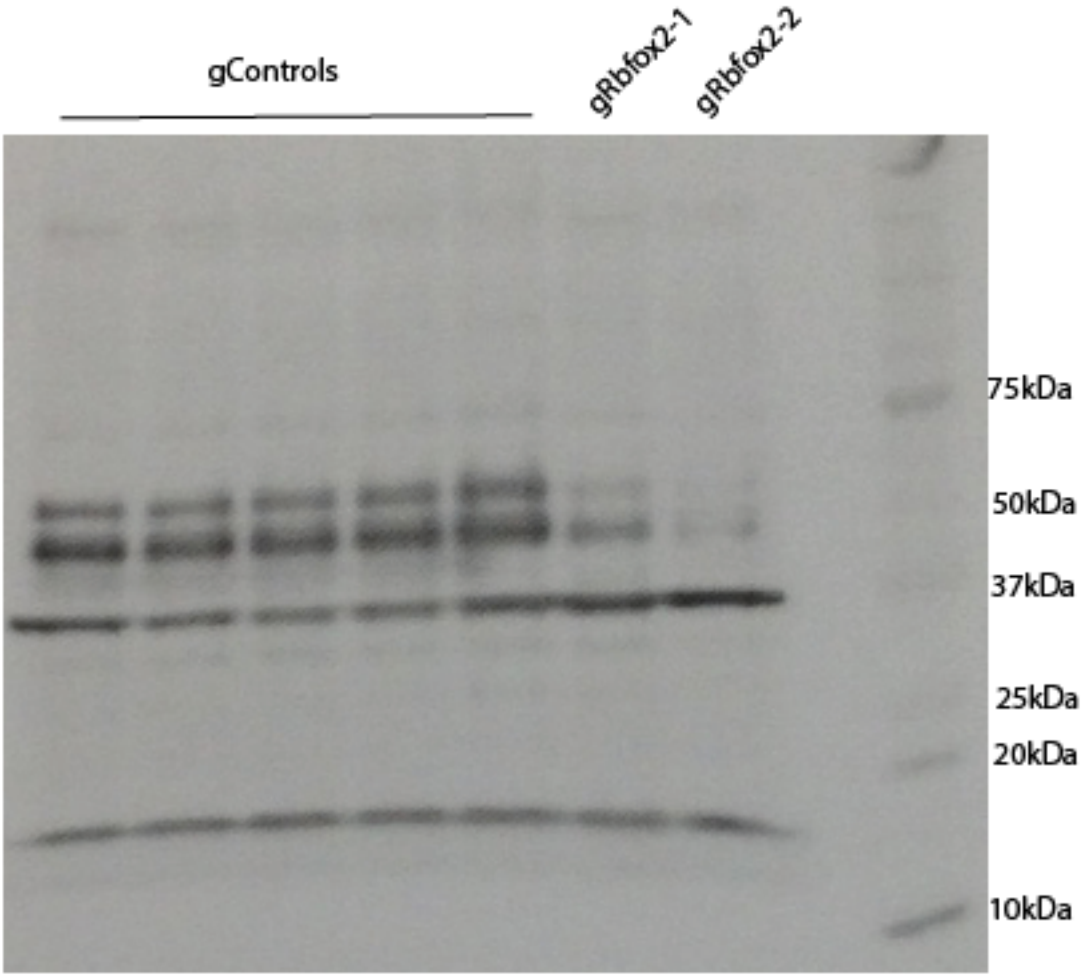

SI Figure 5. CRISPR targeting of Rbfox2*.* Western blot shows lanes from the same gel, with staining for Gapdh and Rbfox2 in cells with infection with Cas9 and guide RNA (control) or with infection of Lenti_v2 containing Cas9 and a guide targeting Rbfox2.

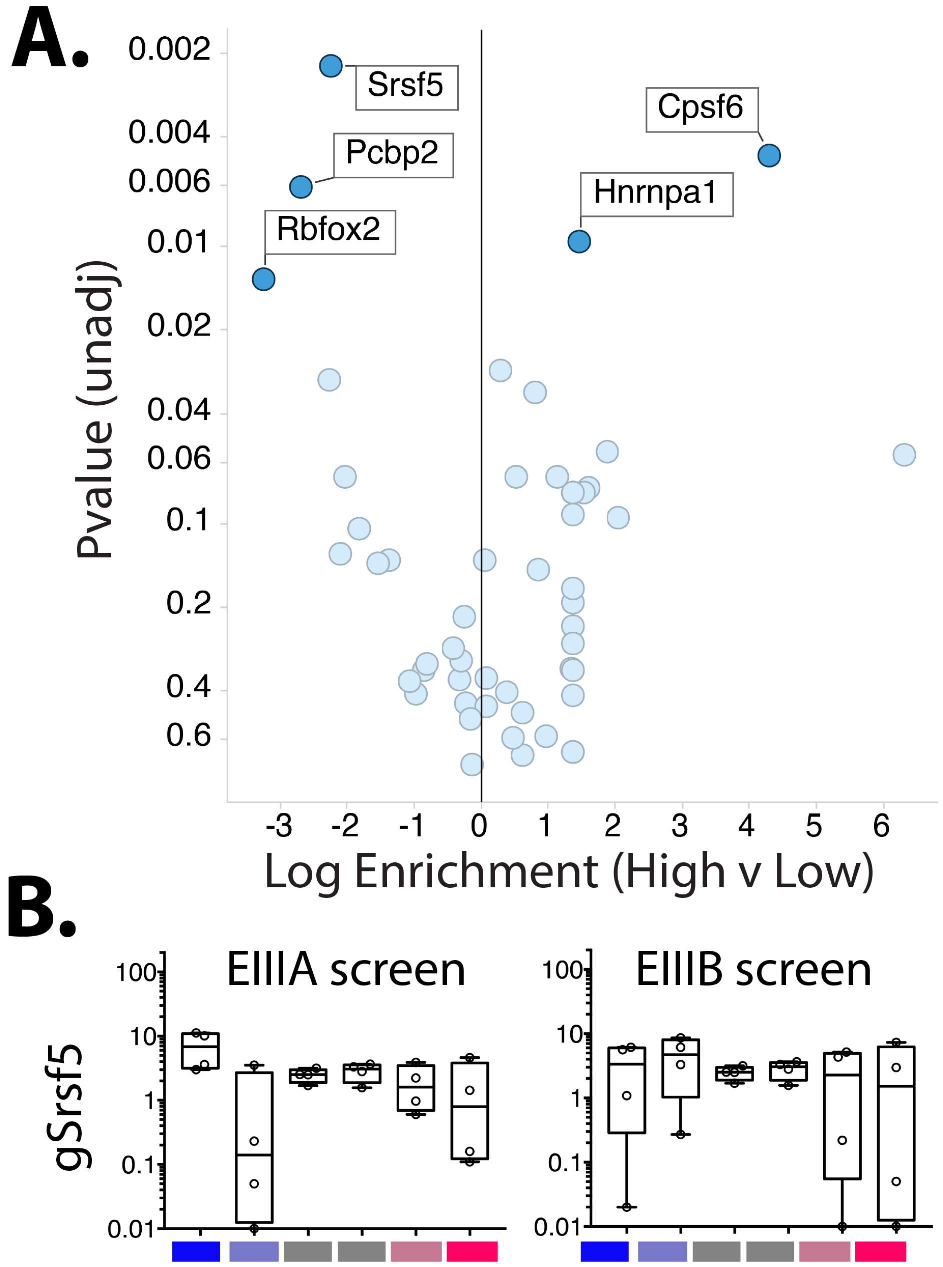

SI Figure 6. Small scale screen for regulators of EIIIA. (A) Volcano plot showing the enrichment of guides for the indicated genes among cells with high levels of EIIIA inclusion versus low levels of EIIIA inclusion. Log enrichment and p-value is from MaGeCK analysis. (B) Plots showing individual guides for Srsf5, and their relative enrichments in sorted cells with low, mid or high levels of EIIIA and EIIIB inclusion (blue to red, respectively).

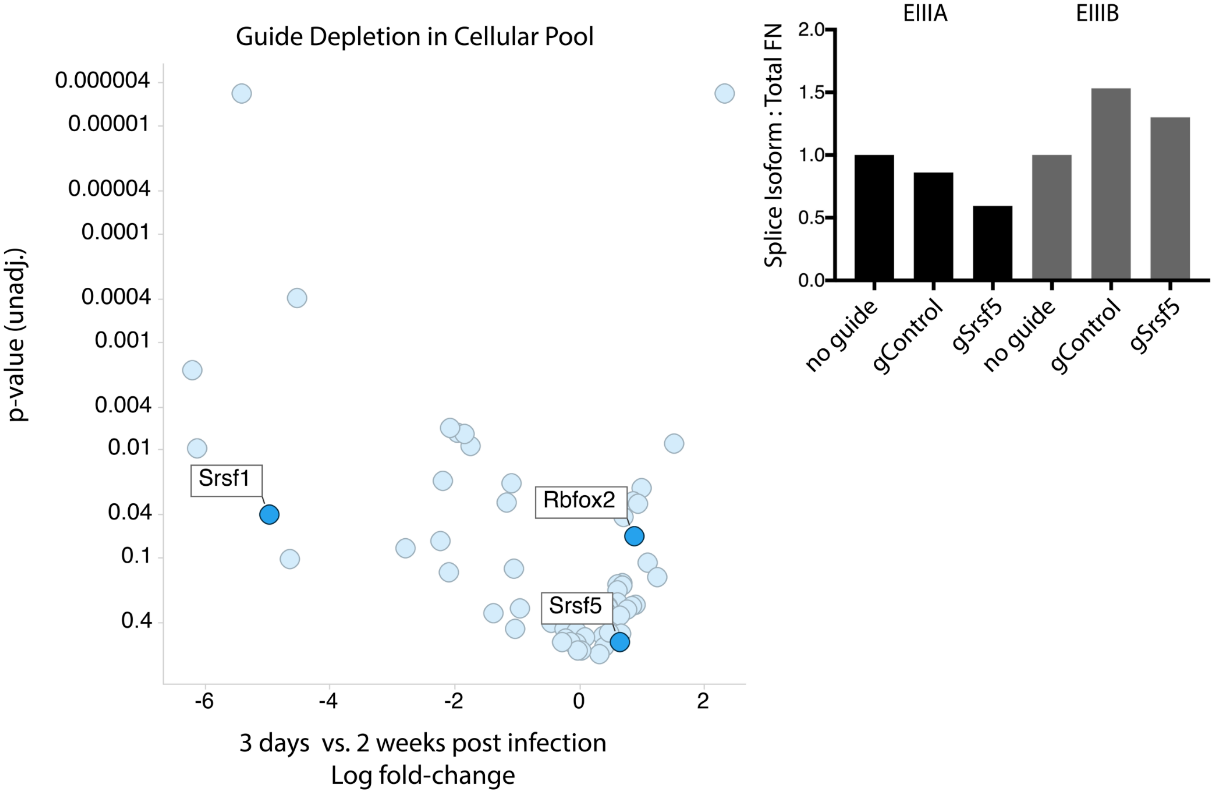

SI Figure 7. Effects of deletion of Srsf1 and Srsf5 on EIIIA splicing. (A) Volcano plot shows the change in guide representation in the cellular pool over time, and p-value by MaGeCK analysis. (B) qPCR analysis of the effect of targeting Srsf5 in cells on EIIIA inclusion level. The level of EIIIA specific PCR product relative to total FN is shown. Cells were not selected on the basis of deletion, so the population will contain some cells without Srsf5 deletion but with gSrsf5 and Cas9.

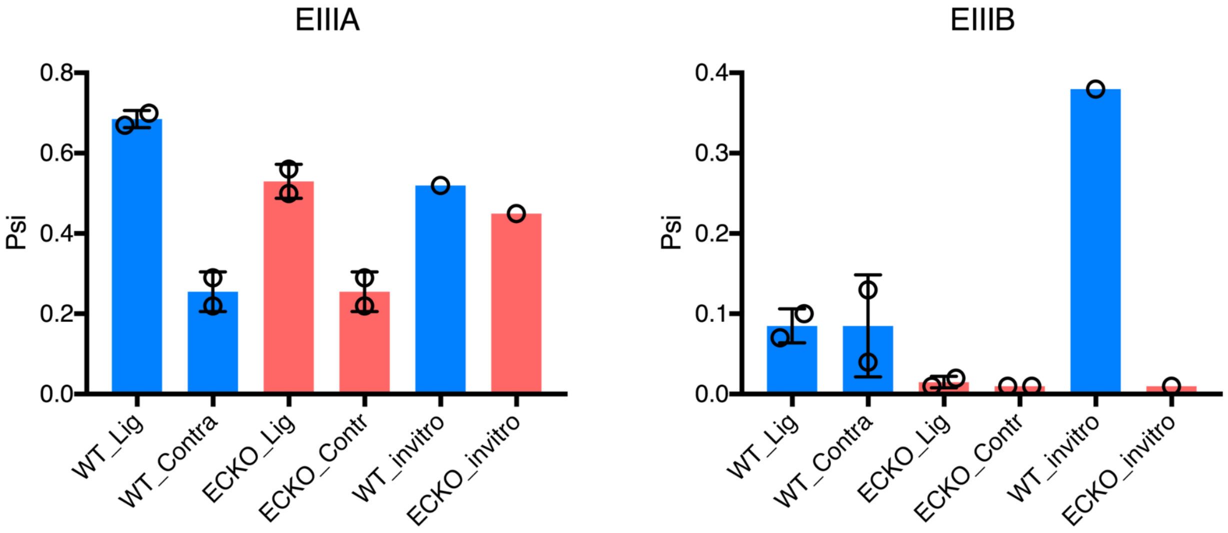

SI Figure 8. Rbfox2 regulates EIIIA and EIIIB in aortic endothelial cells *in vivo* and *in vitro.* Graphs show EIIIA and EIIIB inclusion in FN transcripts by MISO, *in vivo* or *in vitro*. Blue are wild-type controls (Rbfox2 ff) and red are Rbfox2 EC-KO mice (Cdh5(PAC)-CreERT2; Rbfox2 ff). *In vivo* data is from endothelial flush from carotid arteries exposed to low and disturbed flow for 7 days (WT_Lig and ECKO_Lig) or the contralateral arteries exposed to normal flow (WT_Contra and ECKO_Contra). *In vitro* cells were isolated from abdominal aorta and cultured *in vitro* under pro-inflammatory static conditions in the presence of serum for 7 days before sorting AcetylatedLDL+CD31+Icam2+ endothelial cells by flow cytometry.

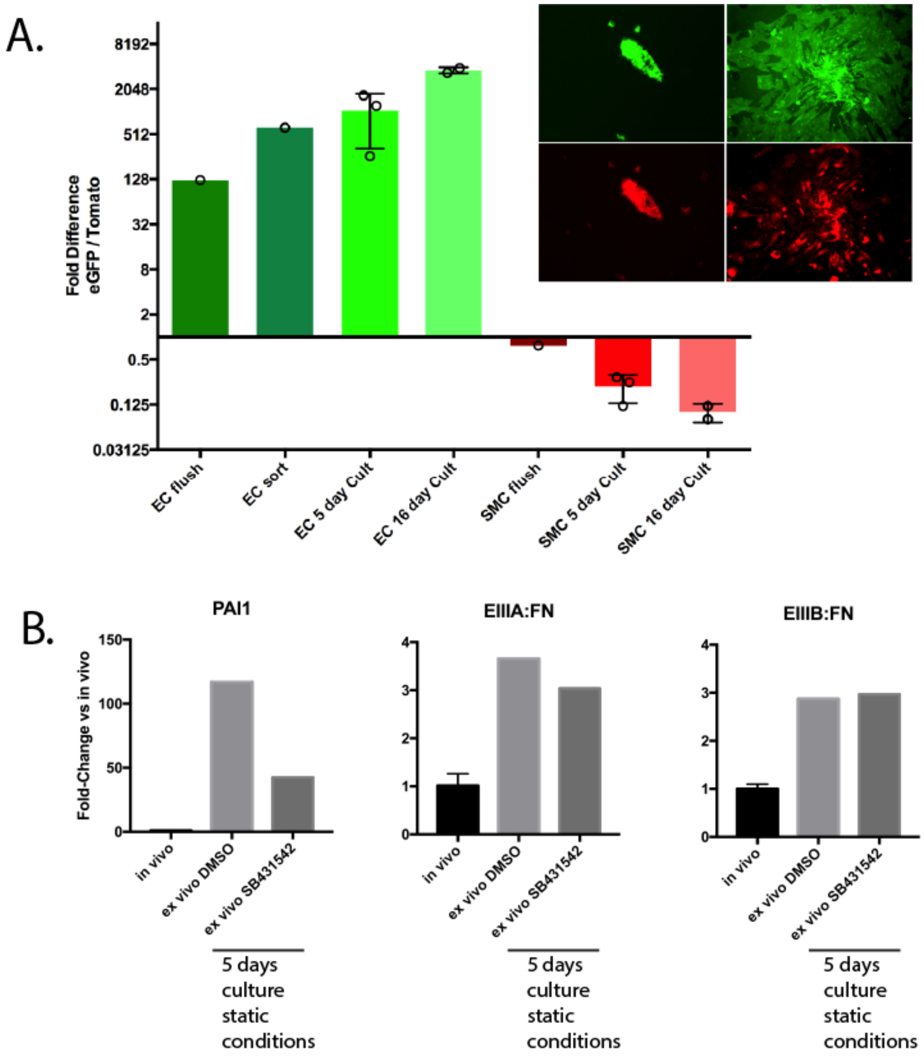

SI Figure 9. Limited effect of TGFb receptor inhibition on EIIIA and EIIIB inclusion in aortic endothelial cells*.* Analysis of mRNA isolated from aortic endothelial cells in vivo, or after 5 days of culture under static conditions in vitro. (A) Cdh5(PAC)-CreERT2; mT/mG mice, were treated with tamoxifen to induce eGFP reporter expression in endothelial cells, and RNA was isolated by Trizol flush of the arterial vessel, or by sorting eGFP+ cells after in vitro culture. Image inset shows typical collagenase flush of intima from artery, which can be purified into endothelial (EC) or non-endothelial (mainly smooth muscle, SMC) by expression of eGFP. Graph shows the expression of eGFP (Cre-induced) vs tdTomato (no Cre) in RNA isolated from intimal artery flush, sorted endothelial cells (eGFP+) or sorted non-endothelial cells (eGFP neg). (B) Cells were cultured in the presence or absence of TGFb receptor (Alk5) inhibitor SB431542 or DMSO control for 5 days. PAI1 is a transcriptional target of TGFb signaling via Alk5. EIIIA:FN and EIIIB:FN refer to the ratio of FN transcripts containing the splice isoforms.

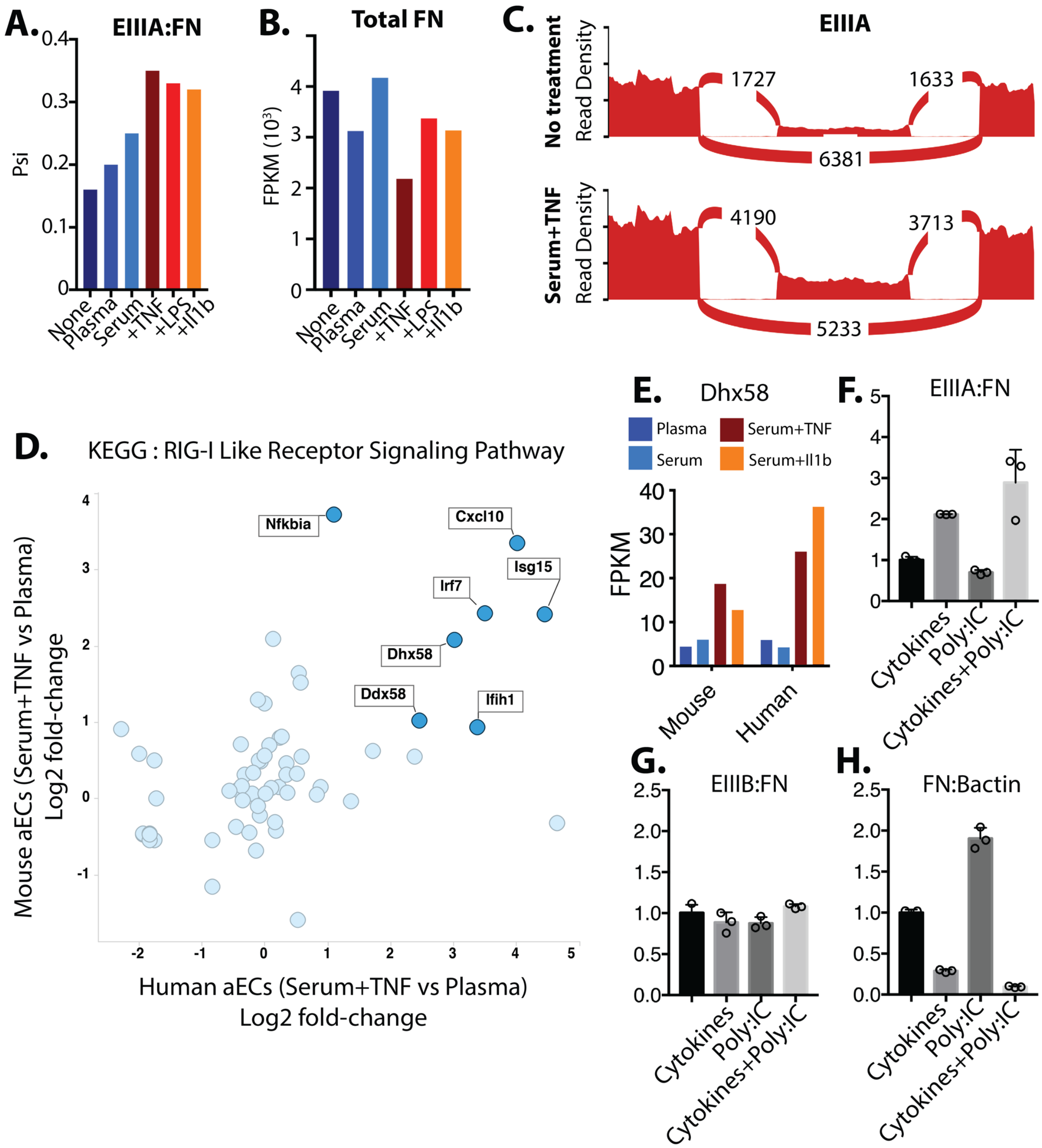

SI Figure 10. Proinflammatory cytokines induce components of the RIG-I-like receptor signaling pathway and EIIIA inclusion. (A-B) Levels of EIIIA inclusion and FN expression from RNA-sequencing data of murine aortic endothelial cells. (A) Psi, or percent splicing in was assessed by MISO. (B) Transcript levels, shown as FPKM or fragments per kilobase of exon per million mapped fragments, were assessed by RSEM. (C) Example plots of EIIIA regulation without treatment and with serum + TNFα treatment. (D) Comparison of the induction of RIG-I like pathway components in mouse and human aortic endothelial cells, treated or not with serum plus TNFα. (E) Transcript levels of Dhx58 in mouse and human aortic endothelial cells with the indicated treatments. (F-H) Quantitative PCR analysis aortic endothelial cells treated for 24hrs with a mixture of TNFα, Il1β, and Infγ, followed by 24hrs of treatment with poly:IC. Graphs show the relative inclusion levels of (F) EIIIA and (G) EIIIB in FN, and total FN transcripts, relative to housekeeping gene β-actin.

SI Table 1. Sorted cell numbers used in each CRISPR screen

Screen	Cell population	Number of Replicates	Cells/Replicate
Targeted (Fn-A)	Lowest 10%	1	9482
Targeted (Fn-A)	Low 10-20%	1	10652
Targeted (Fn-A)	Unsorted	1	~1M
Targeted (Fn-A)	High 10-20%	1	10814
Targeted (Fn-A)	Highest 10%	1	10414
Targeted (Fn-B)	Lowest 10%	1	10583
Targeted (Fn-B)	Low 10-20%	1	15815
Targeted (Fn-B)	Unsorted	1	~1M
Targeted (Fn-B)	High 10-20%	1	10078
Targeted (Fn-B)	Highest 10%	1	10089
Genome-wide (Fn-A)	Low 10%	3	~267,000
Genome-wide (Fn-A)	Mid 20%	6	~775,000
Genome-wide (Fn-A)	High 10%	3	~254,000
Genome-wide (Fn-B)	Low 10%	3	~211,000
Genome-wide (Fn-B)	Mid 20%	6	~653,000
Genome-wide (Fn-B)	High 10%	3	~191,700
